# Entropy as a Tool for the Analysis of Stock Market Efficiency During Periods of Crisis

**DOI:** 10.3390/e26121079

**Published:** 2024-12-11

**Authors:** Daniel Papla, Rafał Siedlecki

**Affiliations:** 1 Department of Insurance, Faculty of Economics and Finance, Wroclaw University of Economics and Business, 53-345 Wroclaw, Poland; 2 Department of Corporate Finance and Public Finance, Faculty of Economics and Finance, Wroclaw University of Economics and Business, 53-345 Wroclaw, Poland; rafal.siedlecki@ue.wroc.pl

**Keywords:** market efficiency, entropy, financial forecasting, economic and financial crisis, random walk

## Abstract

In the article, we analyse the problem of the efficiency market hypothesis using entropy in moments of transition from a normal economic situation to crises or slowdowns in European, Asian and US stock markets and the economy in the years 2007–2023 (2008–2009, U.S. financial sector crises; 2020–2021, Pandemic period; and the 2022–2023 period of Russia’s attack on Ukraine). The following hypothesis is put forward in the article: In periods of economic slowdown and economic crises, the entropy of prices and return rates decreases. According to the principles of physics, in an isolated system, entropy increases and decreases at the moment of external intervention, similar to finance, where during crises and economic slowdowns, there is interference from governments introducing new regulations and intervening in financial markets. The article uses the Shannon entropy method. This measure, as a statistical measure, does not require the assumption of stationarity of time series or a known probability distribution, unlike classical statistical methods. Our results confirm decreased entropy in stock markets during crisis.

## 1. Introduction

The analysis of efficiency and random walk in financial markets is an issue that has been of interest to academics, investment practitioners and market regulators for more than 50 years. The efficient market hypothesis, proposed by Eugene Fama in 1965 [1,2,3] is one of the most debated and researched theories in the social sciences, still leaving much controversy and disagreement about its veracity. According to this hypothesis, a market is considered informationally efficient if all available information is fully reflected in current market prices, meaning there is no possibility of predicting future price changes [2,4]. Today, this topic is still relevant [5]. On the one hand, the development of technology allows easier access to data and information while reducing the time it takes to react to it. However, there is the challenge of selecting and interpreting data in the face of the increasing amount of information available on the different markets [6,7]. In addition, new technologies such as algorithmic trading and artificial intelligence are introducing new elements into the equation, changing market dynamics. With the quick development of new technologies, the market is also creating more complex financial instruments and software that allow transactions to be made without human intervention, e.g., “High-Frequency Traders” [8,9]. The question can then be asked about their efficiency and data selection capabilities. Research on financial markets also raises the important question of whether investors are able to diversify locally and globally through efficient asset allocation, so an important question is whether financial crises actually reduce market efficiency or whether existing statistical methods are sufficiently efficient to analyse this phenomenon [10]. Currently, we are dealing with significant instability not only in the financial markets but also in the economy and social policy (ecology), caused by, among others, the COVID-19 pandemic and the armed conflict in Ukraine and the Middle East, In normal times, the analysis of prices on financial markets indicated the possibility of forecasting long-term trends and the lack of random walks for daily or monthly observations.

In this paper, we address the question of changes in the efficiency of stock markets during periods of crises. The research aims to analyse the efficiency (randomness) of the market during the 2008–2009 and 2020–2022 crisis periods.

However, there are differing opinions on this, as some, such as [11], found no significant changes in market efficiency under the impact of the Asian crisis. Risso [12] showed the lack of effectiveness of efficiency measures based on the Hurst coefficient and Shannon entropy during stock market crashes by analysing cases in such countries as Asia, America and Russia, analysing data from the years 1995 to 2005. According to [10,13,14,15], studying efficiency during the Asian financial crises in 1997 and the global one in 2008, in contrast, found there was a significant decrease in market efficiency during crisis periods. In the case of the 1997 Asian financial crisis, Rizvi and Arshad [10] documented that the weak form of market efficiency was more significant in the pre-crisis period than in the post-crisis period for most East Asian equity markets. Ammy-Driss and Garcin [5] analysed the efficiency of equity markets during COVID-19 using the Hurst exponent and the entropy–Lévy fractional stable motion memory parameter. The authors rejected the efficiency hypothesis based on entropy while obtaining different results with the Hurst exponent. Fox and Sklar, Ball, Malkiel [16,17,18] undertook a discussion of the efficient market hypothesis and its role in financial crises. Fox and Sklar [16] argued that “the efficient market hypothesis (EMH) is responsible for the financial crisis because of an underestimation of the dangers of asset pumping”, while Ball, Malkiel [17,18] believe that blaming the EMH and “statisticians” is overstated and that methods and hypotheses do not influence behaviour in markets. Very interesting results were obtained by [19], who noticed and analysed stock prices in the German and US markets, finding that during periods of stock price declines and crises, entropy (noise variance) decreases, while in moments of return to the pre-crisis situation, information entropy increases, with different markets having different sensitivity to the occurrence of external. Gençay and Gradojevic [20] analysed and compared the dynamics of stock markets during the financial crises of 1987 and 2008. They found that entropy-based analysis can be effective in predicting aggregate market expectations. The authors found that Tsallis entropy was effective for the short and sudden crash, while approximate entropy was a better tool during the prolonged, fundamental crisis of 2008.

The following hypothesis was put forward in the article: In periods of financial and economic slowdown and crises, the entropy of prices and return rates decreases. It means that market efficiency goes from strong to weak. Similar research was conducted by [21,22]. The authors examined the European and American markets in 2007–2009 and 2020–2021 using sample entropy and the symbolic encoding method, partially confirming our hypothesis. In our article, we extended the research to Asian markets and the 2008 financial crisis and used a method more resistant to noise.

According to the principles of physics, in an isolated system, entropy increases and decreases at the moment of external intervention, similar to finance, where during crises and economic slowdowns, there is interference from governments introducing new regulations and intervening in financial markets [23].

To verify the hypothesis and test the efficiency of the capital market, we used the Shannon entropy method to confirm the results. There is also a discussion in the literature about existing methods of analysing market efficiency and their effectiveness under different market conditions. Some studies, such as the work of [7,24,25], point to the usefulness of using entropy and the Hurst exponent in measuring the efficiency of financial markets, while other studies, such as the work of [5,26], suggest the need to take into account additional factors, such as dynamic kurtosis, when analysing market efficiency. It is because “probabilistic prices” in the financial market are characterised by very high volatility and outliers caused by many factors, both measurable and unmeasurable: weather conditions, economic and political factors, technology development [27,28]. In our view, entropy, unlike methods such as autocorrelation tests or linear and nonlinear statistical models, is a better method because it rejects the hypothesis and can also measure the level of randomness of markets [29,30].

Furthermore, it effectively tests information capacity and has a strong foundation in information theory. In the case of medium and strong efficiency, entropy is high [31,32]. At the same time, it decreases in periods of weak efficiency where the market is affected by insider information and external interference (closed system), which is consistent with entropy as a phenomenon dependent on the second law of thermodynamics. In an isolated system or a homogeneous environment, entropy increases, and the system tends to reach the disordered state of maximum entropy more or less slowly. So, we see that these fundamental laws of physics express the natural tendency of systems to a state of chaos [33,34]. In finance and economics, entropy is defined as a measure of the lack of dynamic equilibrium necessary for effective forecasting in financial markets. There is often a view in the literature that in normal times, there is a random walk for daily or monthly prices or return rates [35]; therefore, there is no predictive possibility, i.e., there is a period of high entropy.

## 2. Materials and Methods

Entropy is a measure of the uncertainty of the variables used in statistical phenomena in the context of statistical systems or probability theory.

C.E. Shannon first proposed a measure of uncertainty in 1948 to quantify the amount of “lost information” in phone-line signals mathematically. This measure was later dubbed Shannon entropy. Based on works by [36,37,38], the measure was first presented in his well-known work, “A Mathematical Theory of Communication” [39]. It was a key component in the development of information theory, the first comprehensive mathematical theory of communication.

Shannon made a major contribution when he demonstrated that entropy could be applied to any series in which probabilities exist. This was a major advancement over earlier research by Clausius and Boltzmann, which was limited to thermodynamic systems. The average quantity of “information, choice, and uncertainty” encoded in patterns extracted from a signal or message is what Shannon formally defined as entropy. According to some interpretations, entropy is a system’s degree of disorder and unpredictability. Shannon’s entropy was widely used, especially in finance, and was soon recognised to apply to any series with a well-defined probability distribution.

Consider a categorical random variable with alphabet size *p* and associated cell probabilities *θ*_1_, …, *θ_p_*, with *θ_k_* > 0 and Σ*_k_θ_k_* = 1. This defines the Shannon entropy [39], assumed to be fixed and known as *p*. In this setting, the Shannon entropy in natural units is given by the following:

(1) $$H=-\sum_{k=1}^{p}\theta_{k}ln\left(\theta_{k}\right)$$

Since the probability mass function underlying the situation is unknown in practice, it is necessary to estimate *H* and *θ_k_* from observed cell counts (*y_k_* ≥ 0). The maximum likelihood (ML) estimator, which is created by plugging the ML frequency estimates into the Shannon equation and where $n=\sum_{k=1}^{p}y_{k}$ is the total number of counts, is a particularly straightforward and popular entropy estimator:

(2) $${\hat{\theta }}_{k}^{ML}=\frac{y_{k}}{n}$$

We finally decided on the James–Stein shrinkage estimator [40], which is based on averaging two highly dissimilar models: a lower-dimensional model with more variance but less bias, and a high-dimensional model with low variance and low bias.

(3) $${\hat{\theta }}_{k}^{Shrink}=\lambda t_{k}+\left(1-\lambda \right){\hat{\theta }}_{k}^{ML}$$

where *t_k_* is the shrinkage target and *λ* ∈ [0, 1] is the shrinkage intensity, which can range from 0 (no shrinkage) to 1 (full shrinkage). For *t_k_*, the uniform distribution $t_{k}=\frac{1}{p}$ is a convenient choice.

B.G. statistics, or Boltzmann–Gibbs statistics, is a fundamental concept in modern physics. Extending Clausius’ concept of uncertainty to microscopic states in a system forges a novel connection between statistical mechanics and classical thermodynamics [41]. The B.G. theory does, however, have a narrow range of applications; it is not universal. Boltzmann–Gibbs statistics, according to Tsallis, are only useful for large regimes, or systems devoid of strong correlations and long-range interactions (between microstates). More broadly, extensivity defines a property whose value is proportionate to the size of a system, similar to mass or volume. Because of the strong correlations, or “rules”, that grammar creates between specific words, which limit the number of possible outcomes, the standard BG entropy of coherent sentences in the English language, for example, is non-extensive [42]. But according to Tsallis, entropy is always required to be large because of the basic laws of thermodynamics. To solve the issue of non-extensivity where B.G. entropy fails, Tsallis proposed a generalisation of the Boltzmann–Gibbs entropy in 1988 [43]. He called it non-additive entropy, but others refer to it as Tsallis entropy.

The Rényi entropy, a mathematical generalisation of Shannon entropy that depends on a parameter *q*, was proposed by Alfred Rényi in 1961 [44]. The Central Limit Theorem [45] needed a theoretical proof, which Rényi initially intended to introduce but never finished. Later on, Rényi tried to identify the broadest class of information measures that are consistent with Kolmogorov’s probability axioms and maintain the additivity of statistically independent systems [46]. Nevertheless, Rényi’s entropy was never fully incorporated into statistical mechanics because, unlike Shannon entropy, it has no strong connection to thermodynamics. Because of its connection to free energy, Rényi’s quantity also naturally found a use in physics [47].

A common presumption in many statistical techniques is stationarity. It ensures that under time translations, processes’ mean, variance, and auto-correlation structure stay invariant. For the purpose of modelling and forecasting financial systems, this is especially crucial. However, in the world of finance, series are usually nonstationary, meaning that their statistical characteristics vary over time and they are unpredictable models with drifts and trends. Mathematical operations like logarithms and first differences can make time series almost stationary, making them suitable for use with conventional statistical techniques. Time-dependent methods become good substitutes when transformations to stationary processes are not feasible or possible. For example, time-dependent entropy (TDE) methods have been introduced in information theory to produce a temporal entropy evolution. TDE is able to capture changes in local irregularity in a signal, which can provide valuable information about its nonstationary dynamics [48]. Applications come from a variety of fields, including finance [49,50,51] and physics [52,53,54].

The time-dependent entropy method is defined formally as follows. Let $Z=z_{1},\ldots ,\;z_{n}$ be a nonstationary time series whose statistical characteristics change with time *t*. In a series like this, proper (time-varying) information cannot be captured by standard entropy methods. At each time step $t=0,1,\ldots ,\left[\frac{N}{w}\right]+\left(w-∆-1\right)$ a sliding window $Z_{t}=z_{1+t∆},\ldots ,\;z_{w+t∆}$ of size w≤N is defined with a sliding step of ∆≤w. In order to convert the argument into an integer, use the operator [.]. A temporal evolution of entropy is produced by computing the desired entropy at a specific time *t* using the values of the time series in each window *Zt*. It is well known that variables like window size and time lag can impact outcomes [55]. A few studies using EEG signals are displayed in the Tong paper [56].

The research in the article is based on the analysis of the main world stock market indices (43 countries). The daily data for most of the markets cover the period from 1 January 2007 to 31 December 2023, which were analysed in the form of a one-dimensional set of the stock market index level. We used statistical analysis and estimation, e.g., James–Stein entropy estimator (Hausser, Strimmer 2009). Packages Gretl 2024b and Statistica 13.3 were used for statistical analysis, and for entropy estimation, especially for time-dependent entropy, we used proprietary software created in the R package 4.4.1 for the article. We also conducted a robustness analysis of the received results.

## 3. Results

Analysing our results, we should take into consideration that maximal entropy is in our case 4.6; at this point, there is maximum disorder, and our prediction possibilities are at the lower end. Moreover, due to the nonlinear character of entropy, there is almost no chance of getting to an entropy equal to 0, we consider entropy between 4.0 and 4.3 as medium and below 4.0 as low entropy.

**Figure 1:**
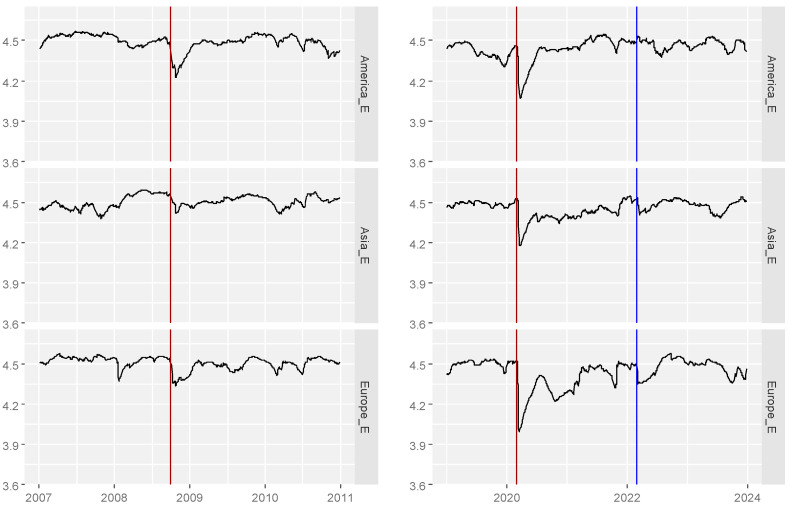
Average time-dependent entropy for America (America_E), Asia (Asia_E) and Europe (Europe_E).

Firstly, we report results for average entropy averaged by continents (Figure 1).

**Figure 2:**
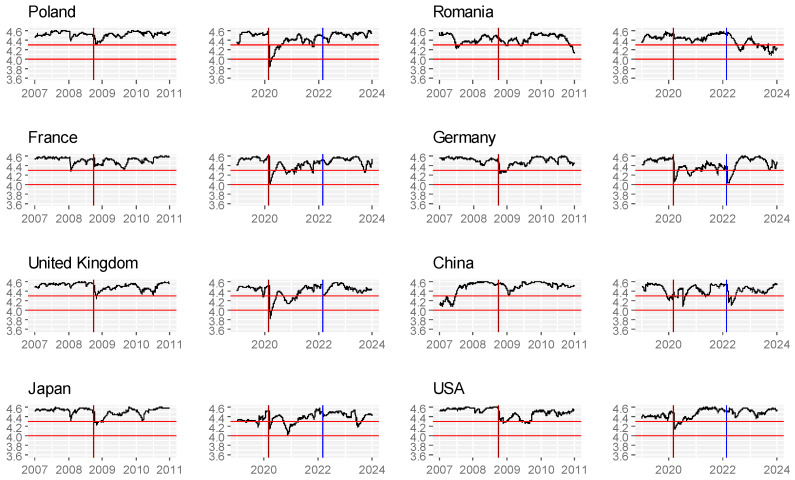
Time-dependent entropy for chosen countries.

Then, we present results for chosen countries (Figure 2).

As we can see, there is one moment in almost all countries, where the entropy falls significantly, this is the beginning of the COVID-19 pandemic. This is especially visible in European countries, but also in the case of the USA or Japan. In this example, only Romania and China differ; in the case of Romania, this could be caused by the specific economic situation of this country. In the case of China, the entropy begins to drop earlier than in other countries, which is related to the fact that the pandemic hit this country first.

## 4. Discussion and Conclusions

In finance, statistical and econometric methods are most often used to analyse relationships and “behaviours”, which require knowledge of the probability distribution and, in the case of prices on financial markets, most often the normal distribution [57]. In our approach, we did not seek the distribution of prices as we had detailed information about them. We have calculated the entropy of systems in different periods. It may be suspected that as statistical mechanics can be deduced from the maximum entropy principle, there will be some analogies between physical systems and the system of prices.

Based on the study for the period 2007–2023, we partially confirmed the hypothesis that in moments of crisis, entropy decreases; that is, market efficiency decreases. Our results partially match the results of [22]. The differences are in the time of occurrence of the entropy decrease. In most of the studied exchanges in 2008–2009, there was an apparent decrease in entropy in most markets, with some of them being small and, interestingly, at different times, while in 2020, it was already significant in all markets. The year 2008 was the moment when the crisis occurred in the United States and, at different times, spread to countries directly or indirectly related to the US financial system, and in many moments, it was an effect based not so much on financial foundations as psychology and the distribution of profits; hence the predictability was lower which is similar to the results obtained by [20]. Unlike 2008, the pandemic period directly affected most countries that had to intervene either in the raw materials or financial markets; hence, according to the law of thermodynamics, entropy decreased (i.e., efficiency decreased) due to external intervention in a closed system. The reaction of the markets to the beginning of the war in Ukraine, which was right after the pandemic period, is fascinating. One could expect another decrease in entropy, which was not very visible. The “defence” mechanism of the markets and the economy probably still existed, and there was no additional (or significant) interference in the markets, which, as a system, again tended to increase entropy.

An increase, i.e., following the period of decline, means that the markets tended towards “random prices”, and thus, one can conclude that it was in the direction of strong efficiency (see Appendix A). So, we can confirm [10,15].

When analysing our results, it is worth looking at the moments of decrease and increase in entropy in different markets and using these methods to analyse the so-called “contagion in financial markets” [58], which is the subject of our further work. It is also worth using other methods of measuring entropy that are more complex, such as permutation entropy [59] or Lévy’s fractional stable motion [5]. An interesting problem is the analysis of the effectiveness of AI and algorithmic trading on markets in periods of high entropy, where they should perform very well, and low entropy, during which, in our opinion, their effectiveness should drop significantly.

## Figures and Tables

**Figure A1:**
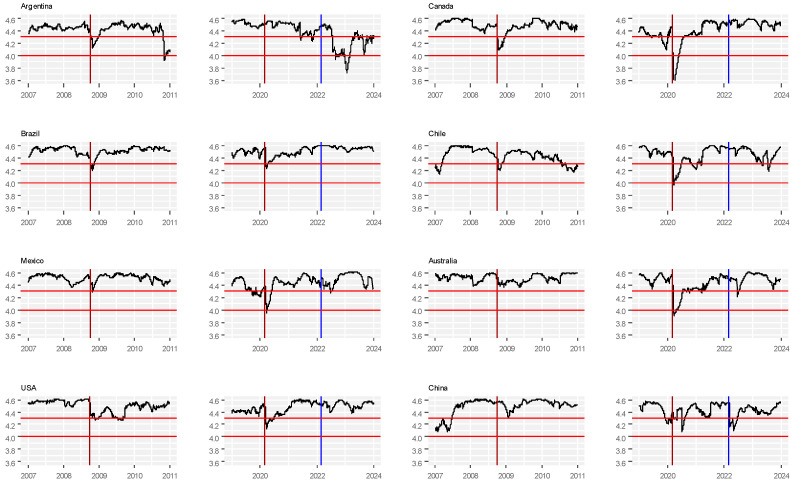

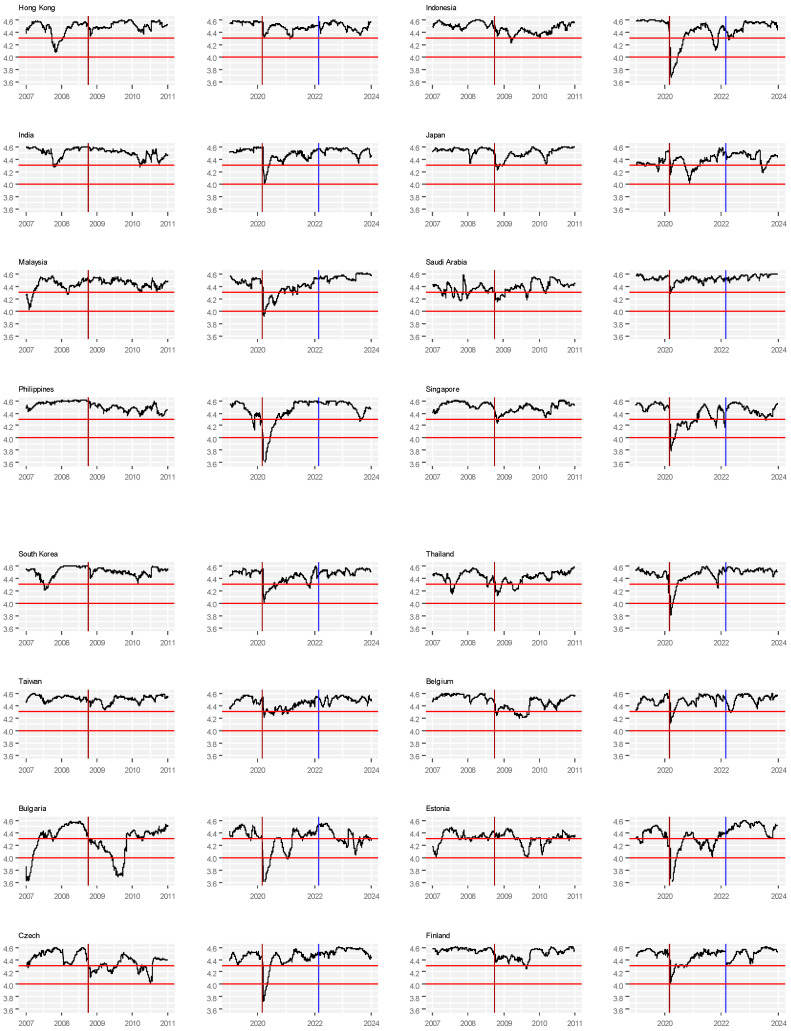

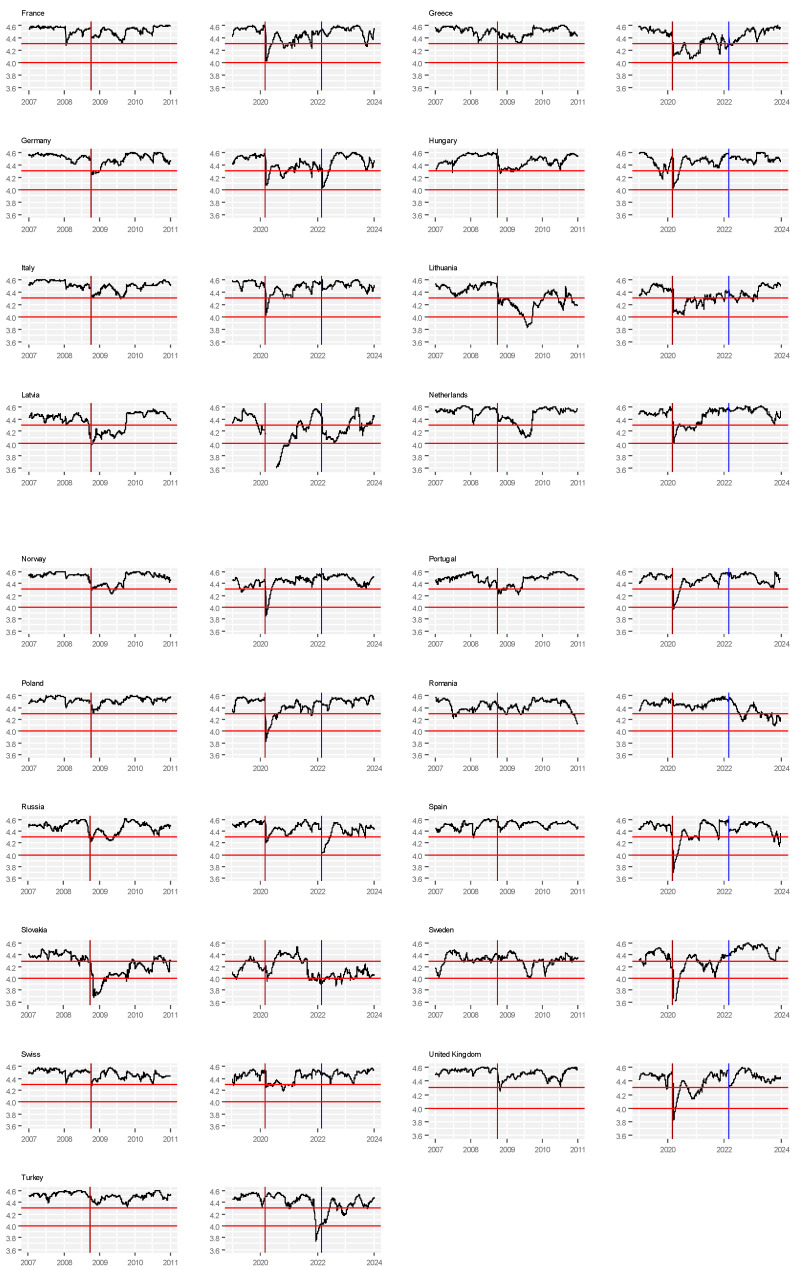
Time-dependent entropy for all countries included in research.

## Data Availability

The raw data supporting the conclusions of this article will be made available by the authors on request.
